# Impact of Medicaid waivers on medication for opioid use disorders in residential facilities: Evidence from twenty-five states

**DOI:** 10.1016/j.drugalcdep.2026.113242

**Published:** 2026-06-20

**Authors:** Pricila H. Mullachery, Dominic Hodgkin, Recai Yucel, Maureen T. Stewart

**Affiliations:** a Temple University, Barnett College of Public Health, Department of Health Services Administration and Policy, Philadelphia, PA, USA; b Brandeis University, Heller School for Social Policy and Management, Waltham, MA, USA; c Temple University, Barnett College of Public Health, Department of Epidemiology and Biostatistics, Philadelphia, PA, USA; d Boston University School of Public Health, Department of Health Law, Policy and Management, Boston, MA, USA

**Keywords:** Medicaid, IMD waiver, Medication for opioid use disorder, Residential treatment

## Abstract

**Background:**

Medicaid Section 1115 waivers allow states to test demonstration programs with the potential to shape access to care for millions of beneficiaries. The Institutions for Mental Diseases (IMD) waiver enables states to use federal funds to treat substance use disorders in residential facilities, bypassing Medicaid’s long-standing IMD exclusion. We assessed the impact of IMD waiver adoption on the use of medication for opioid use disorder (MOUD) by estimating cohort-specific effects among four groups of states that adopted waivers between 2016 and 2019.

**Methods:**

We used Treatment Episode Data Set data. Data on the outcome, i.e., MOUD use among opioid-involved treatment episodes, and covariates were linked to data on waiver effective date, extracted from waiver approval documents. We used difference-in-differences to estimate models stratified by treatment setting (residential vs. non-residential) and adjusted for state-level policy indicators, and state- and year-fixed effects.

**Results:**

MOUD use was low, especially among residential admissions, but increased over time from 5% in 2014–18% in 2019. In adjusted models, the cohort of states that adopted a waiver in 2016 showed higher odds of MOUD use in residential settings (OR=1.49, 95% CI: 1.21–1.84) relative to the comparison group. However, this pattern was not replicated for the later cohorts. Finally, waiver adoption did not have an impact on MOUD use in non-residential settings.

**Conclusion:**

In early adopter states, IMD waivers might have facilitated MOUD use in residential facilities, but their effects varied across cohorts.

## 1. Introduction

The Institutions for Mental Diseases (IMD) waiver provides a pathway for states to expand substance use disorder (SUD) treatment by allowing federal Medicaid dollars to pay for services in psychiatric residential facilities (Glickman and Sisti, 2019). The Medicaid legislation prohibits the use of federal Medicaid funds to pay for services provided to adults in “Institutions for Mental Diseases,” defined as hospitals, nursing facilities, or other facilities with more than 16 beds that primarily offer psychiatric treatment for mental health or substance use disorders (42 U.S.C. § 1396d(i)). This is known as the IMD exclusion, which had the goal of preventing states from shifting the cost of long-term psychiatric care to the federal government (Frank, 2000), and over time, contributed to unequal access to mental health care by restricting Medicaid reimbursement for adults ages 21–64 in inpatient and residential psychiatric facilities (DuBose and Fry-Bowers, 2021).

Estimates suggest that approximately 92% of the 580 psychiatric hospitals nationwide have more than 16 beds, placing them within the IMD definition. Together, these hospitals operate roughly 63,000 beds (Gorman, 2025). In the absence of a waiver, federal Medicaid funds generally cannot be used to cover residential care in these facilities, which may limit access to Medicaid beneficiaries. This highlights the potential for IMD waivers to expand access to this likely underserved population. Individuals with substance use disorders, particularly those with co-occurring physical and mental health conditions, often benefit from short-term treatment in residential facilities before transitioning to outpatient or community-based services (Dams et al., 2024; de Andrade et al., 2019).

Section 1115 of the Social Security Act gives the Secretary of Health and Human Services authority to approve experimental or demonstration projects that promote Medicaid program objectives. Specifically, the IMD Section 1115 waiver allows states to use federal Medicaid funds for residential treatment in IMD facilities. States must apply for waiver approval through the Centers for Medicare & Medicaid Services (CMS). CMS issued two guidance letters related to the IMD waiver, one in 2015 (Centers for Medicare & Medicaid Services, 2015), and a second guidance letter in 2017 (Centers for Medicare & Medicaid Services, 2017), which emphasized requirements to ensure access to medication for opioid use disorder (MOUD). Although MOUD is highly effective, reducing cravings and withdrawal symptoms while lowering the risk of opioid overdose (Mattick et al., 2009, 2014), it remains substantially underutilized, particularly in residential treatment settings (Stahler and Mennis, 2020). Adoption of waivers across a large number of states could be an opportunity to expand access to these medications.

Between 2015 and 2019, 25 states adopted an IMD waiver (Musumeci, 2017). Studies assessing the impact of the waiver have shown mixed results. Early data from nine states suggested that IMD waivers increased the acceptance of Medicaid as a form of payment (Maclean et al., 2021). Single-state studies in California and Virginia showed promising results, such as improved access to SUD treatment and reductions in emergency department visits (Barnes et al., 2020; Bass et al., 2023). But a recent study including 17 states found no impact of the waiver on MOUD use (Lindner et al., 2024).

The present study assessed the impact of IMD waiver adoption on MOUD use by estimating cohort-specific effects among four groups of states that adopted waivers between 2016 and 2019. This design allows us to examine policy effects across adoption cohorts, which may be obscured in pooled analyses. We longitudinally compared states that adopted a waiver at different time points between 2016 and 2019 to a group of states that either (1) adopted a waiver in a later year, or (2) never adopted a waiver during the study period. We also contrasted MOUD utilization between two treatment settings: residential facilities, where we expected the waiver to have a direct impact, and non-residential facilities. In principle, the waiver should not affect treatment delivered in non-residential settings; however, there is potential for spillover effects if mechanisms created to expand MOUD use in residential facilities are disseminated to other settings over time.

## 2. Methods

### 2.1. Sample definition

We used data from the Treatment Episode Data Set (TEDS), which reports data on treatment episodes that occur in facilities that are licensed or certified by state substance use agencies to provide drug or alcohol treatment, and that receive any public funding. We selected opioid-involved treatment episodes, defined as those that recorded opioid use at admission or discharge, or an opioid use disorder diagnosis. We limited the analysis to episodes from 2014 through 2019 to isolate the impact of the waiver from other factors, including the 2020 COVID–19 pandemic that disrupted the healthcare system and the SUPPORT Act that included IMD-related provisions effective starting in October 2019 (U.S. Congress, 2018). We excluded years before 2014 to avoid the pre-Medicaid expansion period.

### 2.2. Outcome

The primary outcome was a binary variable indicating use of medication for opioid use disorder (yes/no), as recorded in TEDS, defined as whether the treatment plan included opioid agonist medications such as methadone or buprenorphine.

### 2.3. Exposure

Exposure to the IMD waiver was defined by the effective date, extracted from waiver approval documents accessed via the Center for Medicare and Medicaid Services (CMS) website. States may include IMD provisions in an existing waiver, as an amendment, or create a new waiver with a focus on substance use disorders. We used the effective date of the amendment or the newly issued waiver that first referred to either the July 2015 State Medicaid Director letter (Centers for Medicare & Medicaid Services, 2015), the updated guidance issued in 2017 (Centers for Medicare & Medicaid Services, 2017), or the provision of treatment for Medicaid enrollees in residential treatment facilities that meet the definition of an Institution for Mental Diseases. CMS waiver approval letters are listed in the Appendix (Supporting Information).

After identifying the effective date of each waiver, states were classified into five groups, or cohorts, based on the calendar year of waiver approval. Using this classification, the 2016 waiver cohort included two states, Massachusetts and Virginia; the 2017 cohort included three states, Maryland, Utah, and New Jersey; the 2018 cohort included ten states, West Virginia, Louisiana, Illinois, Indiana, Kentucky, New Hampshire, Pennsylvania, Vermont, Washington, and Wisconsin; and the 2019 cohort included ten states, Alaska, New Mexico, Rhode Island, Delaware, Kansas, Michigan, Minnesota, Nebraska, North Carolina, and Ohio. We also analyzed data from 21 states that had data available in the TEDS dataset and were never exposed to the waiver during the study period (no waiver group). Table 1 shows sample sizes and states included in each cohort and the “no waiver” group. To define the exposure in the model, we created an indicator, a dummy variable that changes from 0 to 1 in the year the IMD waiver becomes effective. This variable was linked to TEDS data using the state ID variable.

The state of California was excluded from this analysis. Although California was the first state to have a waiver approved in 2015, counties in California adopted the waiver at different times.(Bass et al., 2023) Because TEDS does not allow us to identify county-level treatment episodes, we cannot align treatment admissions with county-specific approval dates. For this reason, all California treatment episodes were excluded from the analysis.

### 2.4. Confounding and covariate selection

To address potential confounding, we adjusted for state-level, time-varying policies plausibly associated with both waiver adoption and MOUD use: (1) Medicaid expansion in the state-year of the treatment episode, obtained from the Kaiser Family Foundation Medicaid Expansion Tracker, (Kaiser Family Foundation, 2025) and (2) drug-related policies, i.e., Good Samaritan Laws and Naloxone Access Laws, from the Prescription Drug Abuse Policy System (PDAPS).(Center for Public Health Law Research, 2025) In addition, we adjusted the models for the following individual-level covariates from TEDS that are associated with MOUD use: age group, binary sex, education, presence of co-occurring mental health conditions, and reporting of other substances (i.e., drugs and alcohol) besides opioids.

### 2.5. Approach

We used a difference-in-differences (DiD) approach adapted to the context of staggered policy adoption. In this approach, we modeled the outcome for each waiver cohort separately. This design offers two advantages: (1) estimating each cohort independently allows us to capture potential differences in policy effect (i.e., heterogeneity) arising from distinct CMS guidance and changing political context at the federal level, and (2) modeling each cohort separately prevents bias from the inclusion of previously exposed (i.e., treated) groups in the comparison group, an issue previously described in the context of staggered adoption (Goodman-Bacon, 2021). By examining each cohort separately, we compare only two groups: one exposed group that adopted a waiver in a particular year, and one comparison group. To avoid including exposed treatment episodes in any given comparison group, we dropped states from the comparison pool after they adopted a waiver, thus removing exposed observations. For example, treatment episodes from states that adopted a waiver in 2016 were dropped from the comparison group used in the 2017 waiver cohort model. Supplementary Table S1 has the list of states in each cohort’s comparison group.

We used logistic regression to model the binary outcome, MOUD use (yes/no), for each cohort. Although DiD traditionally uses linear models, recent work suggests that non-linear models (e.g., logistic, Poisson) are more appropriate for this type of outcome (Wooldridge, 2023). Models were stratified by treatment setting, i.e., residential facilities versus non-residential facilities. Each model included state and year fixed effects, time-varying state-level indicator variables, individual-level covariates, and clustered standard errors at the state level. We used a Markov Chain Monte Carlo procedure to conduct inference via multiple imputation and account for uncertainty due to missing values in individual-level covariates. Odds ratios were obtained by exponentiating the model coefficients.

In a secondary analysis, we re-estimated the odds ratio for MOUD use for each cohort using as a comparison group only states that adopted a waiver in later years during the study period; in other words, we excluded states that were never exposed to the waiver during the study period (no-waiver group). Lastly, as a supplemental analysis, we used Callaway and Sant’anna’s approach (Callaway and Sant’Anna, 2021), implemented via the Stata package CSDID (Rios-Avila et al., 2023), to provide an aggregated estimate and enhance comparability with studies that report pooled effects.

We assessed pre-trends by testing whether trends differed between exposed and comparison states in the pre-adoption period, using an interaction between the cohort-specific treatment indicator and a pre-treatment period indicator.

## 3. Results

**Fig. 1.**
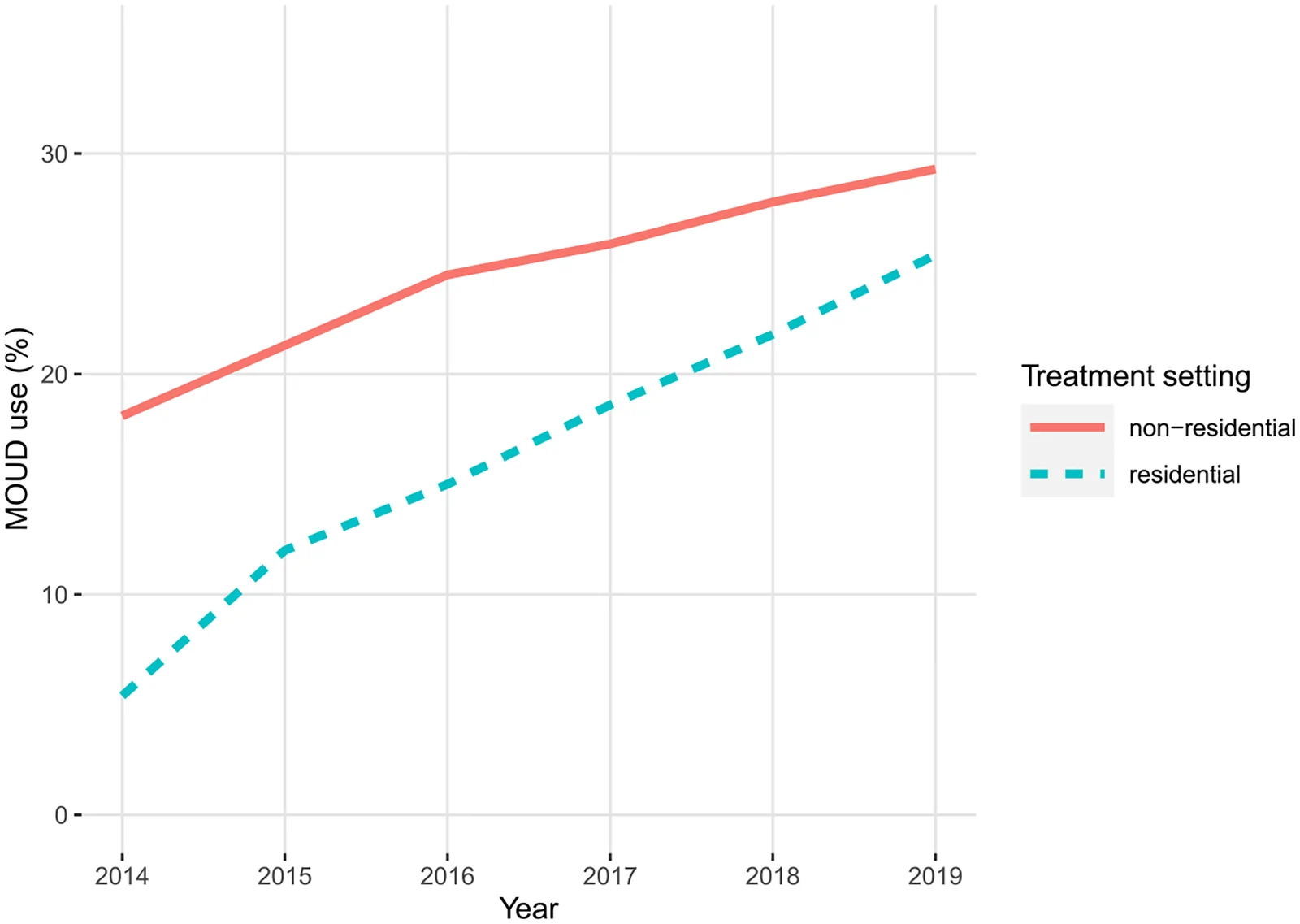
Percent of opioid-involved treatment admissions that included MOUD by treatment setting, TEDS 2014–2019.

Among more than 3 million publicly funded opioid-involved admissions between 2014 and 2019, only 23% had MOUD as part of the treatment. The rate of MOUD use was lower for residential admissions (16.5%) compared to non-residential admissions (24.9%). Between 2014 and 2019, MOUD use increased from 5% to 18% for residential admissions and from 18% to 29% for non-residential admissions (Fig. 1).

Table 2 shows the sample characteristics by waiver cohort and for the group of states that did not adopt a waiver. Individuals aged 25–34 years represented the largest age group, followed by individuals aged 35–44 years. Most admissions were among men, but the proportion of male individuals varied across waiver cohorts, from 67.7% in the 2016 waiver cohort to 53.9% in the 2018 waiver cohort. Individuals with a high school education represented the largest group in the sample, but the distribution by education level varied across the cohorts. For example, the percentage of college-educated individuals ranged from 6.1% in the 2017 cohort to 3.5% in the 2019 cohort. Co-occurring mental health conditions were reported in a significant portion of admissions, ranging from 55.6% in the 2017 cohort to 40.6% in the 2018 cohort. Finally, substances other than opioids were reported in 39.1–34.1% of admissions (Table 2).

Adjusted difference-in-differences models for residential admissions show that states that adopted a waiver in 2016, i.e., 2016 cohort, had higher odds of MOUD use relative to the comparison group. Specifically, admissions in these states had 49% higher odds of MOUD use (OR=1.49, 95% CI: 1.21–1.84) relative to their comparison group. The 2017 cohort also appeared to have higher odds of MOUD use, but this result was not significant at p < 0.05. In contrast, the later cohorts, i.e., 2018 and 2019, had lower odds of MOUD use relative to their comparison group. The 2019 cohort had significantly lower odds of MOUD use (OR=0.71, 95% CI: 0.56–0.90) relative to their comparison group. Finally, results from covariate-adjusted models for non-residential admissions indicate that waivers were not associated with increases in MOUD use, as odds ratios remained close to the null (Table 3).

The secondary analysis, excluding states that were never exposed to the waiver during the study period, showed effects that were larger in magnitude for the 2016 and 2017 cohorts, compared to the alternative specification, with the 2017 cohort now showing significantly higher odds of MOUD use (OR=1.25, 95% CI: 1.04–1.51) relative to the new comparison group, i.e., states that adopted a waiver in later years (Table 4).

A third analytical approach commonly used in the context of staggered adoption(Callaway and Sant’Anna, 2021) showed similar patterns of positive effect of the waiver for early adopting states and negative effect for late adopting states. The overall treatment effect on the treated (ATT) estimate resulting from this approach was not significant, which highlighted the importance of obtaining cohort-specific measures (Supplemental Table S2). Finally, pre-treatment trend tests (Supplemental Table S3) failed to reject the null hypothesis of non-differential trends during the pre-intervention period for cohorts 2016 and 2017, supporting our design. However, significantly different pre-trends were present in cohorts 2018 and 2019.

## 4. Discussion

Data on treatment episodes between 2014 and 2019 showed that only 23% of the episodes included MOUD, with markedly lower use in residential versus non-residential settings. The data also showed that MOUD use increased over the study period. Findings from covariate-adjusted models suggest that IMD waivers may have led to higher MOUD use among treatment episodes occurring in residential facilities, although results were mixed. Specifically, states that adopted an IMD waiver early experienced higher odds of MOUD relative to the comparison group, while states that adopted a waiver later experienced lower odds of MOUD use relative to the comparison group. Finally, waiver adoption did not appear to impact MOUD use in non-residential settings.

Our findings of low MOUD use overall, and particularly low use in residential facilities, are consistent with prior research (Cole et al., 2022; Solomon et al., 2022; Stahler and Mennis, 2020). Low MOUD use has been associated with barriers around provider training (Christine et al., 2024) and insufficient treatment infrastructure (Haffajee et al., 2018). At the system level, efforts to increase MOUD use have included training healthcare providers, reducing prescriber barriers, and expanding the use of telehealth to initiate and manage MOUD (Hailu et al., 2023; Hammerslag et al., 2023). We hypothesized that the IMD waiver would lead to an increase in MOUD use because waivers include provisions that require states to improve the use of evidence-based treatment, aligned with the broader goal of expanding treatment capacity through existing IMD facilities that were previously ineligible for Medicaid reimbursement. Overall, our results suggest that IMD waivers contributed to increased MOUD use in some states.

When restricting the comparison group to states that eventually adopted a waiver, the estimated effects were larger in magnitude for the 2016 and 2017 cohorts, compared to the alternative specification, i.e., including the no-waiver group. This pattern strengthens our conclusions, as future adopters may provide a more appropriate counterfactual given potentially shared unobserved characteristics that may distinguish them from the no-waiver group.

The pattern observed for the early adopters was not replicated for the 2018 and 2019 cohorts. One possible explanation is that higher MOUD use in early adopter cohorts reflects their longer post-adoption observation period, allowing more time for policy effects to emerge. Supplemental Figure S2 presents event-study estimates indicating that increases in MOUD use were more pronounced in later years. In contrast, late adopter states had a shorter follow-up window, ranging from two years to less than one year, for waivers approved in mid- to late 2019. Early adopter states may also have been more motivated, resulting in stronger implementation of the policy and better outcomes.

Our findings add to the literature on the effects of IMD waivers. While these findings are consistent with prior research showing that the IMD waiver is associated with an increased likelihood that residential facilities will offer substance use treatment (Ge et al., 2024), a recent multi-state study reported no overall impact of the waiver on MOUD utilization (Lindner et al., 2024). This finding is consistent with our pooled analysis, which showed no effect of the waiver. Different findings may also reflect underlying differences in data sources, states included, and the analytic strategies used.

Several aspects of our study strengthen confidence in the main findings. Notably, results from models using treatment episodes in non-residential facilities, settings not expected to be affected by the waiver, were not significant. This does not mean that MOUD use did not increase in these settings, but that the increases documented (Fig. 1) were likely related to broad trends, which are accounted for in the DiD model. In addition, the use of DiD helps account for cross-state variation in time-invariant factors, such as provider capacity and local infrastructure, which are unlikely to change in the short term, and nationwide trends over time.

A key methodological limitation of our study is the reliance on the parallel trend assumption, which underlies the difference-in-differences framework. There is evidence of non-parallel trends in the later cohorts. For the early adopters, while pre-treatment trend tests did not reveal significant differences between treatment and comparison groups, un-measured confounding remains a concern. Specifically, unobserved covariates, such as local policies governing access to MOUD in residential settings. Our study aimed to assess the impact of a federal policy—specifically, the IMD waiver—on MOUD use in IMD settings. However, this impact may be underestimated if states in the comparison group implemented other initiatives to promote MOUD uptake, including broad regulations governing facilities licensing and certification independent of the waiver (O’Brien et al., 2022). More granular data are needed to characterize state and local programs and to better understand the extent to which observed increases in MOUD use were driven by federal policy versus independent state or local initiatives.

Moreover, although waivers were similar in intent, there are potential variations in waiver characteristics, particularly related to MOUD provisions, including whether MOUD must be provided on-site or facilitated off-site, types of medications covered, aspects of technical support and enforcement related to changes in CMS administration (E Hinton et al., 2024). States may also vary in the aspects related to the implementation of the waiver. Our findings are limited to the impact of the waiver approval by CMS. Further studies are needed to examine waiver implementation in states.

Misclassification bias is possible because the exposure was defined by the calendar year of waiver adoption rather than the specific approval dates. This may have resulted in misclassification of some pre-waiver treatment episodes as post-waiver, potentially attenuating the estimated effects and biasing results toward the null. In addition, the relatively small number of states in cohorts 2015 and 2016 (totaling five states) could mean that a few states influenced the results in these cohorts. It is possible that unique conditions in some states influenced these results; more in-depth studies examining variation in waiver characteristics across states, as well as their implementation context, are critical to fully understand the factors that drove MOUD use in these early adopting states.

Our data did not allow for the isolation of treatment episodes that took place in IMD facilities. The TEDS classification of residential settings may not perfectly align with the federal definition of IMDs (i.e., residential facilities with more than 16 beds). Because IMD facilities are likely a subgroup of these residential facilities, this may have further attenuated the observed effects. In addition, the TEDS payer variable has substantial and uneven missingness, reaching 100% in some states, which prevents reliable identification of Medicaid-covered admissions; therefore, we did not stratify outcomes by payer. Lastly, because TEDS data does not contain facility IDs, we could not adjust for clustering of treatment episodes within facilities.

.0In conclusion, our findings suggest that IMD waivers led to increased MOUD use in opioid-involved treatment episodes in residential facilities, though this effect was observed only in early adopter states. This heterogeneity of results across cohorts highlights the need to further investigate variation in waiver characteristics that might be underlying these differences. A greater understanding of the extent to which states have implemented IMD waiver requirements, such as ensuring provision of MOUD, as well as the contextual factors shaping implementation, is needed to inform efforts to expand access to high-quality, evidence-based treatment in residential settings.

## Supplementary Material

Supporting Information

## Figures and Tables

**Table 1:** Number of opioid-involved treatment admissions by cohort, TEDS 2014–2019.

Waiver Cohort	Residential	Non-residential
2016 Cohort (MA, VA)	56,226	274,785
2017 Cohort (MD, UT, NJ)	80,306	304,091
2018 Cohort (WV, LA, IL, IN, KY, NH, PA, VT, WA, WI)	118,211	453,791
2019 Cohort (AK, NM, RI, DE, KS, MI, MN, NE, NC, OH)	125,447	595,565
No waiver	320,598	1174,495

“No waiver” jurisdictions were those that did not adopt a waiver during the study period, and included Alabama, Arizona, Arkansas, Colorado, Connecticut, DC, Hawaii, Iowa, Maine, Mississippi, Missouri, Montana, Nevada, New York, North Dakota, Oklahoma, South Dakota, Tennessee, Texas, and Wyoming.

**Table 2:** Characteristics of treatment episodes among people with opioid use reported by groups according to IMD waiver year (cohort).

	2016 Waiver Cohort	2017 Waiver Cohort	2018 Waiver Cohort	2019 Waiver Cohort	No waiver
Age groups					
12–17	0.5	0.4	1.0	0.6	0.8
18–24	13.4	14.2	15.1	13.3	14.3
25–34	46.8	41.1	45.7	45.7	40.8
35–44	24.0	21.4	22.6	23.7	22.1
45–54	11.5	16.2	10.8	11.3	14.8
55 +	3.8	6.7	4.8	5.4	7.2
Sex					
Male	67.7	63.1	53.9	58.8	64.7
Female	32.3	36.9	46.1	41.2	35.3
Education					
No schooling to high school incomplete	26.5	25.7	27.9	24.5	25.6
High school complete	46.6	56.5	49.1	46.8	47.8
Some college	21.8	11.8	19.3	25.2	21.6
College or more	5.1	6.1	3.7	3.5	5.0
Co-occurring mental health condition					
No	56.0	44.4	59.4	54.9	49.7
Yes	44.0	55.6	40.6	45.1	50.3
Substances other than opioids					
No	71.2	65.9	61.3	65.3	60.9
Yes	28.8	34.1	38.7	34.7	39.1

**Table 3:** Odds ratio of medication for opioid use disorder (MOUD) use by cohort and type of treatment setting, TEDS 2014–2019.

IMD waiver adoption year	Odds ratio of MOUD use					
	Residential			Non-residential		
	OR	95% CI	n	OR	95% CI	n
2016 cohort	1.49	(1.21–1.84)	567,199	1.05	(0.87–1.28)	2240,793
2017 cohort	1.07	(0.87–1.31)	567,816	0.82	(0.69–1.03)	2246,953
2018 cohort	0.59	(0.36–0.99)	569,491	0.8	(0.48–1.30)	2221,286
2019 cohort	0.71	(0.56–0.90)	558,001	1.1	(0.90–1.24)	2234,561

Notes: Odds ratios were obtained by exponentiating the coefficients from difference-in-differences models containing treatment episodes for each cohort (exposed) and a comparison group composed of treatment episodes not exposed to the waiver in each cohort. Comparison states include those not yet exposed and those never exposed to the waiver. Treatment episodes exposed to the waiver over time were dropped from the comparison group. For example, episodes included in the 2016 cohort were dropped from the comparison group used in the 2017 cohort model. Models included individual-level covariates (sex, age, presence of co-occurring mental health conditions, and other substance use), time-varying state-level indicator variable for Medicaid expansion, Good Samaritan, and Naloxone Laws, state- and year-fixed effects, and clustered standard errors at the state level.

**Table 4:** Odds ratio of medication for opioid use disorder (MOUD) use by cohort and type of treatment setting, excluding states that never adopted a waiver during the study period, TEDS 2014–2019.

IMD waiver adoption year	Odds ratio of MOUD use					
	Residential			Non-residential		
	OR	95% CI	n	OR	95% CI	n
2016 cohort	1.91	(1.36–2.67)	255,834	1.12	(0.74–1.70)	1106,356
2017 cohort	1.25	(1.04–1.51)	262,613	0.82	(0.49–1.37)	1112,516
2018 cohort	0.89	(0.61–1.29)	258,126	0.70	(0.37–1.31)	1086,849
2019 cohort	-	-	-	-	-	-

Note: comparison states included only those that were eventually exposed between 2015 and 2019. As such, there was no comparison group for the 2019 cohort, since all jurisdictions had been exposed in previous years. Models included the same covariates as in Table 3.

## Appendix A. Supporting information

Supplementary data associated with this article can be found in the online version at doi:10.1016/j.drugalcdep.2026.113242.
