# Supplementary material for: Impact of Medicaid waivers on medication for opioid use disorders in residential facilities: Evidence from twenty-five states
Source: Drug Alcohol Depend. Author manuscript; Available in PMC 2026 Aug 8. (PMC13449180; doi:10.1016/j.drugalcdep.2026.113242)
Supplement: Supplementary file 1 [file NIHMS2193292-supplement-Supporting_Information.pdf]

Supporting Information from original article: Mullachery, P. H., Hodgkin, D., Yucel, R., & Stewart, M. (2026). Impact of Medicaid Waivers on Medication for Opioid Use Disorders in Residential Facilities: Evidence from Twenty-five States. *Drug and Alcohol Dependence*, 113242.

Supplemental Tables and Figures:

Supplemental Table S1: Jurisdictions (states + DC) included in comparison groups for each cohort.

Cohort	Jurisdictions in comparison group
2016 waiver cohort	Alabama*, Alaska, Arizona*, Arkansas*, Colorado*, Connecticut*, DC*, Delaware, Hawaii*, Illinois, Indiana, Iowa*, Louisiana, Kansas, Kentucky, Maine*, Maryland, Michigan, Minesota, Mississippi*, Missouri*, Montana*, Nebraska, Nevada*, New Hampshire, New Jersey, New Mexico, New York*, North Carolina, North Dakota*, Pennsylvania, Ohio, Oklahoma*, Rhode Island, South Dakota*, Tennessee*, Texas*, Utah, Vermont, Washington, West Virginia, Wisconsin, Wyoming*
2017 waiver cohort	Alabama*, Alaska, Arizona*, Arkansas*, Colorado*, Connecticut*, DC*, Delaware, Hawaii*, Illinois, Indiana, Iowa*, Louisiana, Kansas, Kentucky, Maine*, Michigan, Minesota, Mississippi*, Missouri*, Montana*, Nebraska, Nevada*, New Hampshire, New Mexico, New York*, North Carolina, North Dakota*, Pennsylvania, Ohio, Oklahoma*, Rhode Island, South Dakota*, Tennessee*, Texas*, Vermont, Washington, West Virginia, Wisconsin, Wyoming*
2018 waiver cohort	Alabama*, Alaska, Arizona*, Arkansas*, Colorado*, Connecticut*, DC*, Delaware, Hawaii*, Iowa*, Kansas, Maine*, Michigan, Minesota, Mississippi*, Missouri*, Montana*, Nebraska, Nevada*, New Mexico, New York*, North Carolina, North Dakota*, Ohio, Oklahoma*, Rhode Island, South Dakota*, Tennessee*, Texas*, Wyoming*
2019 waiver cohort	Alabama*, Arizona*, Arkansas*, Colorado*, Connecticut*, DC*, Hawaii*, Iowa*, Maine*, Mississippi*, Missouri*, Montana*, Nevada*, New York*, North Dakota*, Oklahoma*, South Dakota*, Tennessee*, Texas*, Wyoming*

*Episodes from these jurisdictions were dropped in the secondary analysis, which excluded the "no waiver" group

Supplemental Figure S1: Trend in MOUD by waiver cohort, TEDS 2014-2019

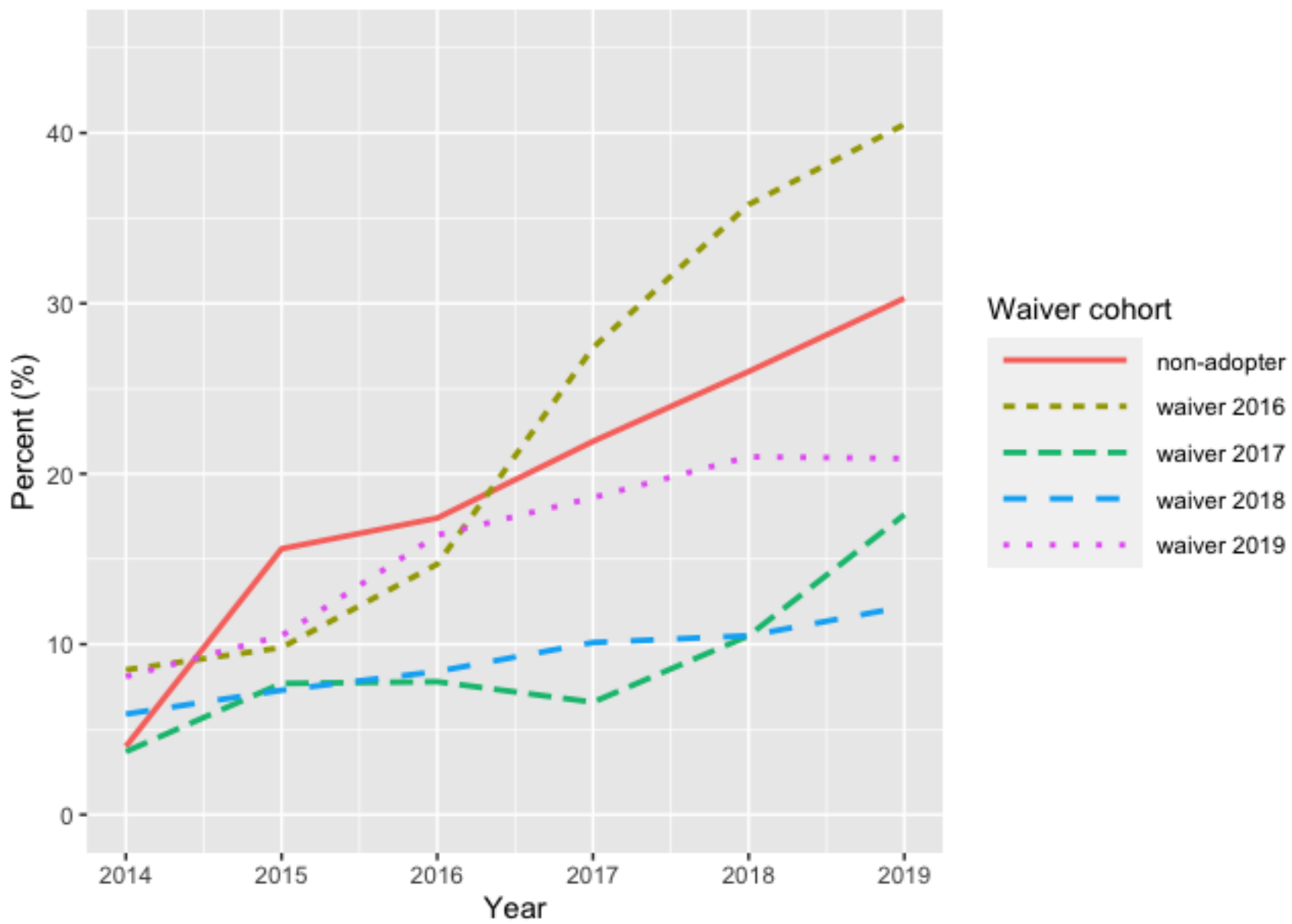

Supplemental Table S2: Coefficients from models using the approach by Calaway and Santana, TEDS 2014-2019

	Residential		Non-residential	
	Coefficient	95% CI	Coefficient	95% CI
Overall ATT	-0.005	(-0.104;0.094)	-0.005	(-0.041;0.031)
2016 cohort ATT	0.141	(0.101;0.181)	0.028	(-0.008;0.064)
2017 cohort ATT	-0.064	(-0.192;0.065)	0.014	(-0.038;0.066)
2018 cohort ATT	-0.072	(-0.118;-0.027)	-0.031	(-0.093;0.031)
2019 cohort ATT	-0.066	(-0.099;-0.034)	-0.04	(-0.084;0.004)

Note: We used the Stata package CSDID, which estimates cohort- and time-specific treatment effects (group-time ATTs). We compared each treated cohort to units that have not been treated (not yet treated + never treated). To obtain overall treatment effects, CSDID aggregates group-time ATTs using weights proportional to the cohort size. This produces an overall average treatment effect on the treated (ATT).

Supplemental Figure S2: Event study plot from models in Table S2, TEDS 2014-2019

(A) Residential

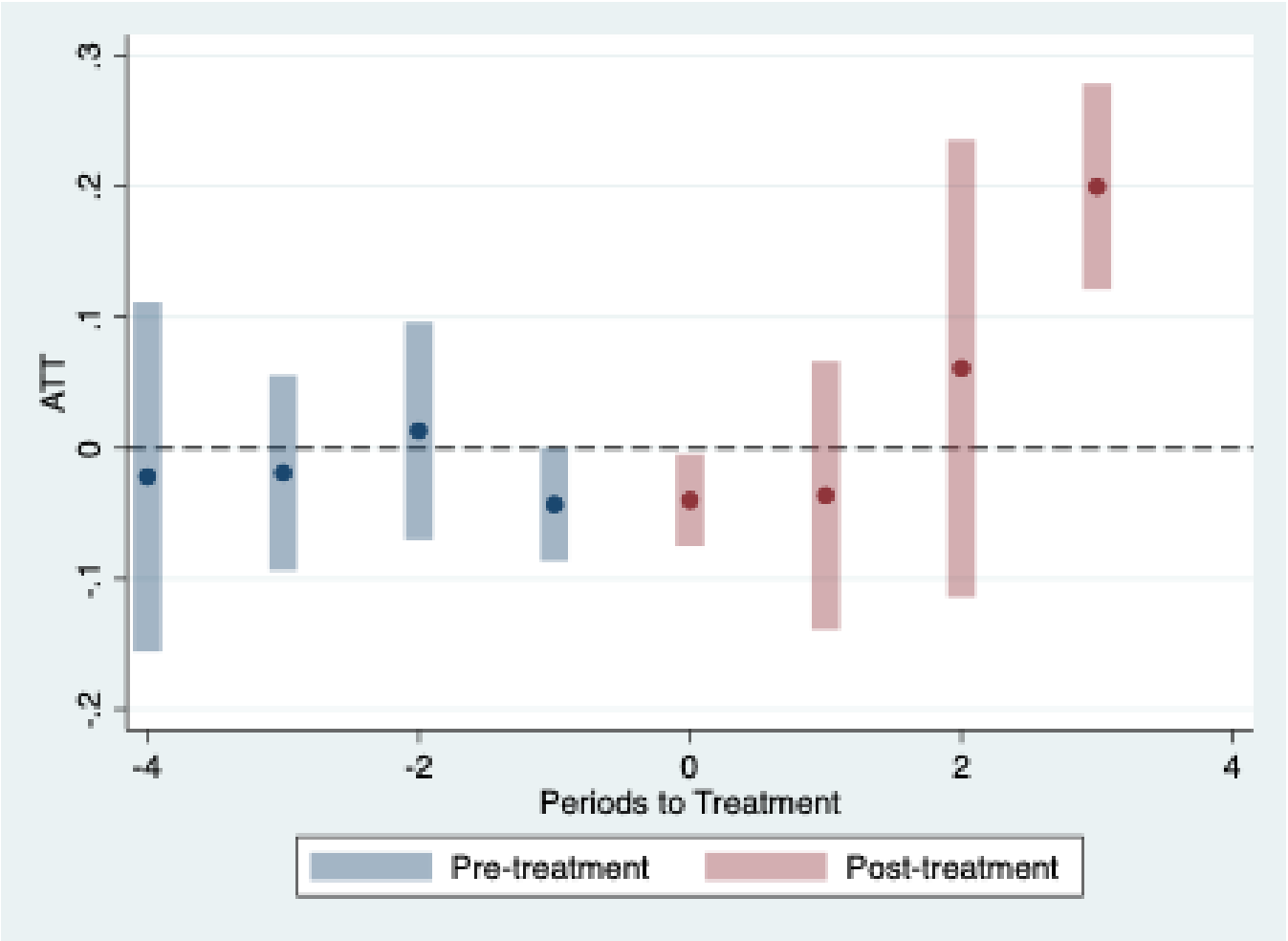

(B) Non-residential

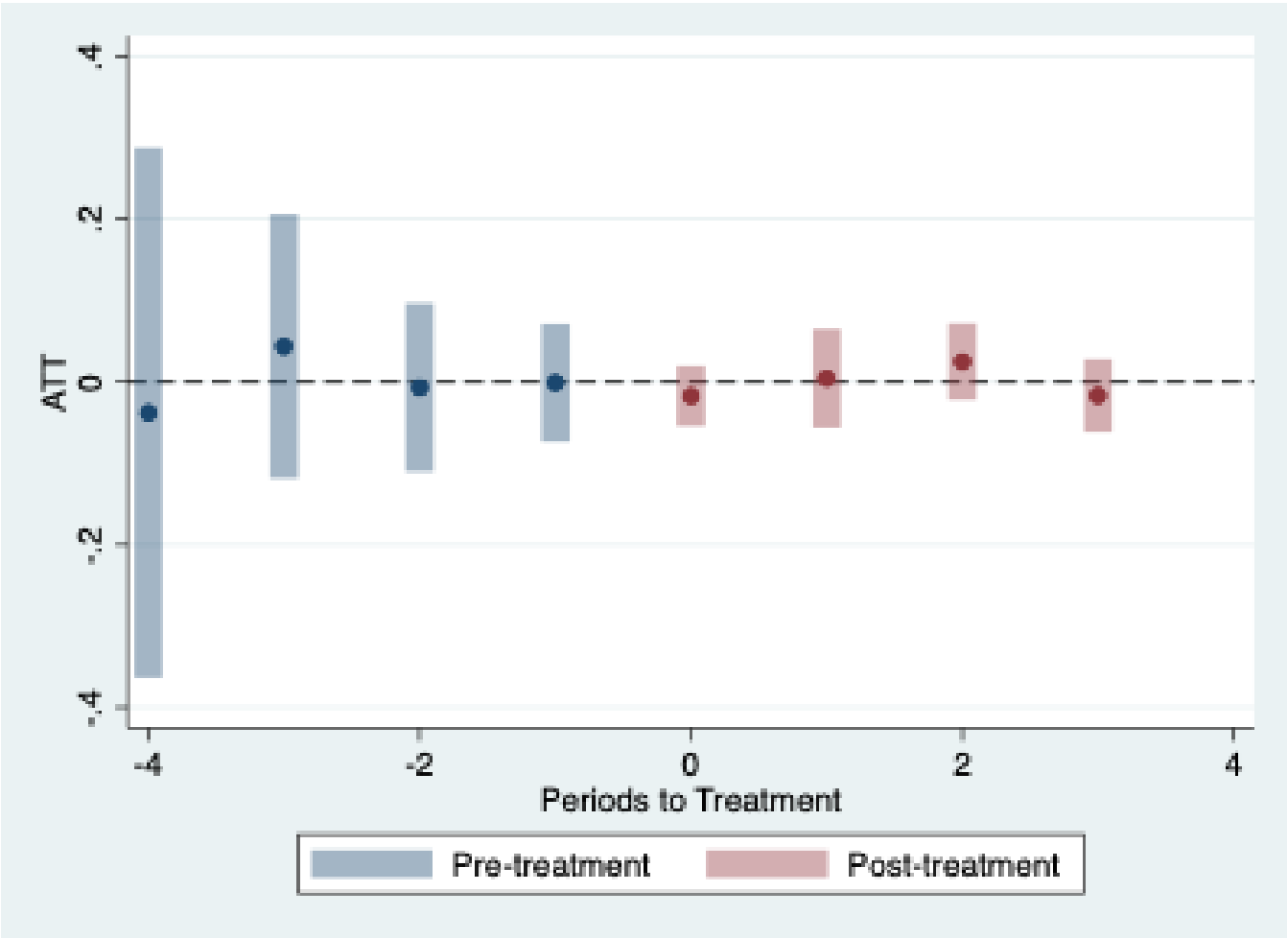

Note: Results are from the CSDID event study plot using models in Supplemental Table S2

Supplemental Table S3: Pre-treatment parallel trend test

Group/cohort	Interaction Coefficient (pre-exposure time dummy * treated dummy)	Robust standard error	p-value
2016 waiver cohort	0.251715	0.313352	0.422
2017 waiver cohort	0.114356	0.085061	0.179
2018 waiver cohort	0.909846	0.239762	<0.001
2019 waiver cohort	0.597063	0.157438	<0.001

Note: Models included individual-level covariates (sex, age, presence of co-occurring mental health conditions, and other substance use), time-varying state-level indicator variable for Medicaid expansion, Good Samaritan, and Naloxone Laws, state- and year-fixed effects, and clustered standard errors at the state level.

Appendix: Section 1115 IMD Waivers

State	Waiver documentation	Reference to IMD Waiver or CMS 2015 or 2017 guidance regarding the IMD waiver	Effective date
Massachusetts	Waiver: MassHealth<br><br>Approval: https://www.medicaid.gov/Medicaid-CHIP-Program-Information/By-Topics/Waivers/1115/downloads/ma/MassHealth/ma-masshealth-stcs-11042016.pdf<br><br>Project description: https://www.medicaid.gov/Medicaid-CHIP-Program-Information/By-Topics/Waivers/1115/downloads/ma/MassHealth/ma-masshealth-demo-amend-ext-app-08302016.pdf	"MassHealth and the Department of Public Health's (DPH) Bureau of Substance Abuse Services have collaborated on the development of a Substance Use Disorder 1115 demonstration proposal. This proposal has been developed in response to the July 27, 2015 letter from CMS to State Medicaid Directors titled *New Service Delivery Opportunities for Individuals with a Substance Use Disorder*."	Nov 4, 2016
Virginia	Waiver: The Virginia Governor's Access Plan (GAP) and Addiction and Recovery Treatment Services (ARTS) Delivery System Transformation<br><br>Approval: https://www.medicaid.gov/Medicaid-CHIP-Program-Information/By-Topics/Waivers/1115/downloads/va/Governors-Access-Plan-GAP/va-gov-access-plan-gap-appvl-amdmnt-12152016.pdf	"This amendment authorizes the state to strengthen Virginia's substance use disorder (SUD) delivery system to improve the care and health outcomes for Virginia Medicaid beneficiaries with SUD consistent with the CMS guidance issued in the July 27, 2015 State Medicaid Director letter on new service delivery opportunities for individuals with SUD (see SMD # 15- 003)."	Dec 15, 2016
Maryland	Waiver: HealthChoice<br><br>Approval: https://www.medicaid.gov/Medicaid-CHIP-Program-Information/By-Topics/Waivers/1115/downloads/md/Health-Choice/md-healthchoice-ext-appvl-122016.pdf	"The HealthChoice section 1115 demonstration extension will allow the Maryland Department of Health and Mental Hygiene (DHMH) to implement initiatives that will serve individuals with substance use disorders (SUD) consistent with the CMS guidance issued in the July 27, 2015 State Medicaid Director letter on new service delivery opportunities for individuals with SUD (see SMD #15-003)."	Jan 1, 2017
Utah	Waiver: Utah Primary Care Network	"Utah is seeking a five-year extension of the Primary Care Network Demonstration Waiver	Nov 1, 2017

	Approval:<br>https://www.medicaid.gov/Medicaid-CHIP-Program-Information/By-Topics/Waivers/1115/downloads/ut/Primary-Care-Network/ut-primary-care-network-cms-ext-apprvl-10312017.pdf<br><br>Project description:<br>https://www.medicaid.gov/Medicaid-CHIP-Program-Information/By-Topics/Waivers/1115/downloads/ut/ut-primary-care-network-pa.pdf	pursuant to Section 1115(f) of the Social Security Act. The State is requesting several amendments to its existing 1115 waiver. They are as follows: (…) Waiver of the Medicaid Institutions for Mental Diseases (IMD) exclusion under Section 1905(a)(29)(B) of the Social Security Act to allow for medically necessary Residential Treatment Services for individuals with substance use disorders."	
New Jersey	Waiver: "NJ Family Care Comprehensive Demonstration<br><br>Approval:<br>https://www.medicaid.gov/Medicaid-CHIP-Program-Information/By-Topics/Waivers/1115/downloads/nj/Comprehensive-Waiver/nj-1115-request-cms-amend-apprvl-oud-sud-program-11012017.pdf	"This demonstration authorizes New Jersey to expand the OUD/SUD benefit package to combat prescription drug abuse and opioid use disorders, and provide treatment services, including withdrawal management services in residential treatment facilities that meet the definition of an Institution for Mental Disease (IMD)."	Oct 21, 2017
West Virginia	Waiver: Evolving West Virginia Medicaid's Behavioral Health Continuum of Care<br><br>Approval:<br>https://www.medicaid.gov/Medicaid-CHIP-Program-Information/By-Topics/Waivers/1115/downloads/wv/continuum-care/wv-creating-continuum-care-medicaid-enrollees-substance-creating-continuum-sud-stcs-20171010.pdf<br><br>Project description:<br>https://www.medicaid.gov/Medicaid-CHIP-Program-Information/By-Topics/Waivers/1115/downloads/wv/continuum-	"Objective 2: Increase enrollee access to, and utilization of, appropriate SUD treatment services based on American Society of Addiction Medicine (ASAM) criteria. (…) • Make residential treatment opportunities available to Medicaid beneficiaries, as appropriate."<br><br>July 2015 State Medicaid Director Letter. "New Service Delivery Opportunities for Individuals with a Substance Use Disorder." Centers for Medicare & Medicaid Services.	Jan 1, 2018

	care/wv-creating-continuum-care-medicaid-enrollees-substance-demo-app-12062016.pdf		
Louisiana	Waiver: Healthy Louisiana OUD/SUD Demonstration<br><br>Approval: https://www.medicaid.gov/medicaid/section-1115-demonstrations/downloads/la-healthy-oud-sud-org-demo-appvl-02012018.pdf	"This SUD demonstration authorizes Louisiana to receive federal financial participation (FFP) for the continuum of services to treat addiction to opioids or other substances, including services provided to Medicaid enrollees with substance use disorder residing in certain residential treatment facilities that meet the definition of an Institution for Mental Disease (IMD)."	Feb 1, 2018
Illinois	Waiver: Illinois Healthcare Transformation<br><br>Approval: https://www.medicaid.gov/Medicaid-CHIP-Program-Information/By-Topics/Waivers/1115/downloads/il/Behavioral-Health-Transformation/il-behave-health-transform-demo-apprvl-05072018.pdf	"This approval authorizes the state to receive federal financial participation (FFP) for the continuum of services to treat addictions to opioids and other substances. including services provided to Medicaid enrollees with a substance use disorder (SUD) who are short-tern residents in residential and inpatient treatment facilities that meet the definition of an Institution for Mental Diseases (lMD)."	Jul 1, 2018
Indiana	Waiver: Health Indiana Plan<br><br>Approval: https://www.medicaid.gov/Medicaid-CHIP-Program-Information/By-Topics/Waivers/1115/downloads/in/Healthy-Indiana-Plan-2/in-healthy-indiana-plan-cms-amend-appvl-02012018.pdf	"The SUD program contributes to a comprehensive statewide strategy to combat prescription drug abuse and opioid use disorders, and expands the SUD benefits package to cover short-term residential services for all Medicaid enrollees."	Feb 1, 2018
Kentucky	Waiver: Kentucky Helping to Engage and Achieve Long Term Health (KY HEALTH)<br><br>Approval: https://www.medicaid.gov/Medicaid-CHIP-Program-Information/By-Topics/Waivers/1115/downloads/ky/health/ky-health-cms-appvl-011218.pdf	"The KY HEALTH demonstration will also include a substance use disorder (SUD) program available to all Kentucky Medicaid beneficiaries to ensure that a broad continuum of care is available to Kentuckians with SUD. (…) As part of the SUD initiative, the state may receive FFP for the continuum of (…) to treat OUD and other SUDs that are provided to all Medicaid beneficiaries in an IMD as authorized by this demonstration."	Jan 12, 2018

New Hampshire	Waiver: Substance Disorder Treatment and Recovery Access – TRA)<br><br>Approval: https://www.medicaid.gov/Medicaid-CHIP-Program-Information/By-Topics/Waivers/1115/downloads/nh/sud-treatment-recovery-access/nh-sud-treatment-recovery-access-stcs-07102018.pdf	"This approval authorizes New Hampshire to receive federal financial participation (FFP) for the provision of all Medicaid state plan services-including a continuum of services to treat addictions to opioids and other substances-for Medicaid enrollees primarily diagnosed with opioid use disorder (OUD) and/or other substance use disorders (SUD) who are short-term residents in residential and inpatient treatment facilities that meet the definition of an Institution for Mental Diseases (lMD)."	Jul 10, 2018
Pennsylvania	Waiver: Pennsylvania Medicaid Coverage for Former Foster Care Youth from a Different State and Substance Use Disorder (SUD) demonstration.<br><br>Approval: https://www.medicaid.gov/medicaid/section-1115-demonstrations/downloads/pa-former-foster-care-youth-diff-state-amend-appvl-sud-demo-20180628.pdf	"This approval authorizes Pennsylvania to receive federal financial participation (FFP) for the continuum of services to treat addictions to opioids and other substances, including services provided to Medicaid enrollees with SUD who are short-term residents in residential and inpatient treatment facilities that meet the definition of an Institution for Mental Diseases (IMD) and who receive services via managed care."	Jul 1, 2018
Vermont	Waiver: Vermont Global Commitment to Health<br><br>Approval: https://www.medicaid.gov/Medicaid-CHIP-Program-Information/By-Topics/Waivers/1115/downloads/vt/Global-Commitment-to-Health/vt-global-commitment-to-health-cms-appvl-demo-amend-06062018.pdf	"This approval authorizes the state to receive federal financial participation (FFP) for the continuum of services to treat addictions to opioids and other substances, including services provided to Medicaid enrollees with a substance use disorder who are short-term residents in residential and inpatient treatment facilities that meet the definition of an Institution for Mental Diseases (IMD)."	Jun 6, 2018
Washington	Waiver: Medicaid Transformation Project<br><br>Approval: https://www.medicaid.gov/Medicaid-CHIP-Program-Information/By-Topics/Waivers/1115/downloads/wa/medicaid-transformation/wa-medicaid-transformation-CMS-amendment-request-apprvl-07172018.pdf	"This approval authorizes Washington to receive federal financial participation (FFP) for the provision of all Medicaid state plan services-including a continuum of services to treat addictions to opioids and other substances-for Medicaid enrollees primarily diagnosed with opiate use disorder (OUD) and/or other substance	Jul 17, 2018

		use disorders (SUD) who are short-term residents in residential and inpatient treatment facilities that meet the definition of an Institution for Mental Diseases (IMD)."	
Wisconsin	Waiver: BadgerCare Reform<br><br>Approval: https://www.medicaid.gov/medicaid/section-1115-demonstrations/downloads/wi-badgercare-reform-cms-ext-appvl-10312018.pdf	"In addition, this demonstration will also include a substance use disorder (SUD) program (described in STCs 26–32) available to all Wisconsin Medicaid beneficiaries. The SUD program contributes to a comprehensive statewide strategy to combat prescription drug abuse and opioid use disorders and expands the SUD benefits package to cover short-term residential services in facilities that qualify as institutions for mental diseases (IMDs) for all Medicaid enrollees."	Oct 31, 2018
Alaska	Waiver: Alaska Behavioral Health Reform<br><br>Approval: https://www.medicaid.gov/medicaid/section-1115-demonstrations/downloads/ak-behavioral-health-demo-benefits-amend-appvl-09032019.pdf	""The SUD component of this demonstration authorizes Alaska to receive federal financial participation (FFP) for the provision of all Medicaid state plan and approved demonstration services-including a continuum of services to treat addiction to opioids and other substances-for Medicaid enrollees who are primarily receiving treatment and withdrawal management services and are short-term residents in residential and impatient treatment facilities that meet the definition of an Institution for Mental Diseases (IMD)."	Jan 1, 2019
New Mexico	Waiver: New Mexico Centennial Care 2.0<br><br>Approval: https://www.medicaid.gov/Medicaid-CHIP-Program-Information/By-Topics/Waivers/1115/downloads/nm/Centennial-Care/nm-centennial-care-appvl-ltr-12142018.pdf	"This approval authorizes the state to receive federal financial participation (FFP) for the continuum of services to treat addictions to opioids and other substances, including services provided to Medicaid enrollees diagnosed with a substance use disorder (SUD) who are short-term residents in residential and inpatient treatment facilities that meet the definition of an Institution for Mental Diseases (IMD)."	Jan 1, 2019

Rhode Island	Waiver: Rhode Island Comprehensive Demonstration<br><br>Approval: https://www.medicaid.gov/Medicaid-CHIP-Program-Information/By-Topics/Waivers/1115/downloads/ri/Comprehensive-Demonstration/ri-global-consumer-choice-compact-cms-ext-request-appvl-12202018.pdf	"In addition, this approval authorizes the state to receive federal financial participation (FFP) for the continuum of services to treat addictions to opioids and other substances, including services provided to Medicaid enrollees with SUD who are short-term residents in residential and inpatient treatment facilities that meet the definition of an Institution for Mental Diseases (IMD)."	Jan 1, 2019
Delaware	Waiver: Delaware Diamond State Health Plan<br><br>Approval: https://www.medicaid.gov/medicaid/section-1115-demonstrations/downloads/de-dshp-ext-appvl-07312019.pdf	"This approval authorizes Delaware, once CMS approves the state's SUD implementation plan, to receive federal financial participation (FFP) for expenditures for otherwise covered services furnished to otherwise eligible individuals who are primarily receiving treatment and withdrawal management services for substance use disorder who are short-term residents in facilities that meet the definition of an Institution for Mental Diseases (lMD)."	Aug 1, 2019
Kansas	Waiver: KanCare<br><br>Approval: https://www.medicaid.gov/Medicaid-CHIP-Program-Information/By-Topics/Waivers/1115/downloads/ks/KanCare/ks-kancare-cms-appvl-sud-implementation-plan-20190807.pdf	"…with this letter, the state may begin receiving Federal Financial Participation for Kansas Medicaid recipients residing in the Institutions for Mental Disease setting under the terms of this demonstration"	Aug 7, 2019
Michigan	Waiver: Pathways to Integration<br><br>Approval: https://www.medicaid.gov/Medicaid-CHIP-Program-Information/By-Topics/Waivers/1115/downloads/mi/Pathway-to-Integration/mi-pathway-integration-initial-appvl-20190405.pdf	"This approval authorizes Michigan to receive federal financial participation (FFP) for the provision of all Medicaid state plan services, including a continuum of services to treat substance use disorder for Medicaid beneficiaries primarily diagnosed with opioid use disorder (OUD) and/or other SUD who are short-term residents in residential and inpatient treatment facilities that meet the definition of an Institution for Mental Diseases (lMD)."	Apr 5, 2019

Minnesota	Waiver: Minnesota Substance Use Disorder System Reform<br><br>Approval: https://www.medicaid.gov/Medicaid-CHIP-Program-Information/By-Topics/Waivers/1115/downloads/mn/substance-use-disorder-system/mn-sud-reform-demo-appvl-20190628.pdf	"Approval of this demonstration will allow Minnesota to test a new way to strengthen the state's behavioral health care system by increasing opportunities for substance use disorder (SUD) services provided to patients within Institutions for Mental Disease (IMDs)."	Jul 1, 2019
Nebraska	Waiver: Nebraska Substance Use Disorder<br><br>Approval: https://www.medicaid.gov/medicaid/section-1115-demonstrations/downloads/ne-sud-demo-appvd-sud-implementation-plan-20190709.pdf	"…with this letter, the state may begin receiving Federal Financial Participation for services provided to Nebraska Medicaid recipients residing in Institutions for Mental Disease for the treatment of a SUD"	Jul 9, 2019
North Carolina	Waiver: North Carolina Medicaid Reform Demonstration<br><br>Approval: https://www.medicaid.gov/Medicaid-CHIP-Program-Information/By-Topics/Waivers/1115/downloads/nc/Medicaid-Reform/nc-medicaid-reform-demo-demo-appvl-20181019.pdf	"This approval authorizes the state to receive federal financial participation (FFP) for the continuum of services to treat addictions to opioids and other substances, including provided to Medicaid enrollees with a SUD who are short-term residents in residential and inpatient treatment facilities that meet the definition of an Institution for Mental Diseases (IMD)."	Jan 1, 2019
Ohio	Waiver: Ohio Section 1115 Demonstration Waiver for Substance Use Disorder Treatment<br><br>Approval: https://www.medicaid.gov/medicaid/section-1115-demonstrations/downloads/oh-substance-use-disorder-treatment-demo-appvl-09242019.pdf	"Approval of this demonstration will allow Ohio to receive federal financial participation (FFP) for the provision of all Medicaid state plan services, including a continuum of services to treat addictions to opioids and other substances for 1 otherwise eligible Medicaid beneficiaries primarily diagnosed with opioid use disorder (OUD) and/or SUD who are short-term residents in residential and inpatient treatment facilities that meet the definition of an Institution for Mental Diseases (IMD)."	Oct 1, 2019
